# Breast cancer risk factors and mammographic density among 12518 average-risk women in rural China

**DOI:** 10.1186/s12885-023-11444-7

**Published:** 2023-10-09

**Authors:** Huijiao Yan, Wenhui Ren, Mengmeng Jia, Peng Xue, Zhifang Li, Shaokai Zhang, Lichun He, Youlin Qiao

**Affiliations:** 1 School of Population Medicine and Public Health, Chinese Academy of Medical Sciences and Peking Union Medical College, Beijing, 100730 China; 2 National Cancer Center/National Clinical Research Center for Cancer/Cancer Hospital, Chinese Academy of Medical Sciences and Peking Union Medical College, Beijing, 100021 China; 3 Changzhi Medical College, Changzhi, 046000 Shanxi China; 4 Department of Cancer Epidemiology, The Affiliated Cancer Hospital of Zhengzhou University & Henan Cancer Hospital, Henan Engineering Research Center of Cancer Prevention and Control, Henan International Joint Laboratory of Cancer Prevention, Zhengzhou, 450008 China; 5 Mianyang Maternal & Child Health Care Hospital, Mianyang Children’s Hospital, Mianyang, 621000 China

**Keywords:** Mammographic density, Risk factor, Breast cancer

## Abstract

**Background:**

Mammographic density (MD) is a strong risk factor for breast cancer. We aimed to evaluate the association between MD and breast cancer related risk factors among average-risk women in rural China.

**Methods:**

This is a population-based screening study. 12518 women aged 45–64 years with complete MD data from three maternal and childcare hospitals in China were included in the final analysis. ORs and 95%CIs were estimated using generalized logit model by comparing each higher MD (BI-RADS b, c, d) to the lowest group (BI-RADS a). The cumulative logistic regression model was used to estimate the *OR*_*trend*_ (95%CI) and *P*_*trend*_ by treating MD as an ordinal variable.

**Results:**

Older age (OR_trend_ = 0.81, 95%CI: 0.79–0.81, per 2-year increase), higher BMI (OR_trend_ = 0.73, 95%CI: 0.71–0.75, per 2 kg/m2), more births (OR_trend_ = 0.47, 95%CI: 0.41–0.54, 3 + vs. 0–1), postmenopausal status (OR_trend_ = 0.42, 95%CI: 0.38–0.46) were associated with lower MD. For parous women, longer duration of breastfeeding was found to be associated with higher MD when adjusting for study site, age, BMI, and age of first full-term birth (OR_trend_ = 1.53, 95%CI: 1.27–1.85, 25 + months vs. no breastfeeding; OR_trend_ = 1.45, 95%CI: 1.20–1.75, 19–24 months vs. no breastfeeding), however, the association became non-significant when adjusting all covariates. Associations between examined risk factors and MD were similar in premenopausal and postmenopausal women except for level of education and oral hormone drug usage. Higher education was only found to be associated with an increased proportion of dense breasts in postmenopausal women (OR_trend_ = 1.08, 95%CI: 1.02–1.15). Premenopausal women who ever used oral hormone drug were less likely to have dense breasts, though the difference was marginally significant (OR = 0.54, *P* = 0.045). In postmenopausal women, we also found the proportion of dense breasts increased with age at menopause (OR_trend_ = 1.31, 95%CI: 1.21–1.43).

**Conclusions:**

In Chinese women with average risk for breast cancer, we found MD was associated with age, BMI, menopausal status, lactation, and age at menopausal. This finding may help to understand the etiology of breast cancer and have implications for breast cancer prevention in China.

**Supplementary Information:**

The online version contains supplementary material available at 10.1186/s12885-023-11444-7.

## Background

Breast cancer is an important public health problem, and has become the most frequently diagnosed malignancy worldwide. The incidence rate in Asia is relatively lower compared with that in Western countries, but the gap is narrower and 45.4% of new breast cancer cases are diagnosed in Asia countries since it is the most populous continent [1]. Breast cancer is a complex disease with various etiological causes. Nonmodifiable factors known to increase the risk of breast cancer include age, family history of breast cancer [2], reproductive factors [3] and genetic mutation [4]. Modifiable risk factors include postmenopausal obesity [5], alcohol consumption [6] and physical inactivity [7].

Apart from those mentioned above, mammographic density (MD) is a strong risk factor for breast cancer. MD represents the percentage of radiologically dense area of breast, which, comprised of more stromal and epithelial tissues, appears white on mammography images [8]. Previous studies have constantly found that women with 75% or greater percent of MD had four to six times higher risk of breast cancer compared with women with density in less than 10% [9, 10]. MD is modifiable and decreased with age [11]. Other modifiable and nonmodifiable factors were found to be associated with MD, such as alcohol consumption [12], body mass index (BMI) [13], dietary factors [14], reproductive factors [15]. This suggested many of risk factors that increased breast cancer risk might work through their effects on MD. Breast dense tissues also diminished the capacity of detecting breast cancers by mammogram and thus increase the risk of interval breast cancer between screening tests [16].

Asian women had smaller breast size, smaller absolute breast dense volume, higher percent breast density compared to Caucasian women [17–19]. The Breast Imaging Reporting and Data System (BI-RADS) divided MD into four categories based on quantitative assessment, which provided the risk for developing breast cancer to clinicians [20]. The BI-RADS density classification was frequently used for MD assessment in breast cancer screening programs in China and the worldwide. Asian women were more frequently reported to have heterogeneously or extremely dense breasts than Caucasian women. Prior studies have evaluated associations between MD and breast cancer related risk factors in Chinese women. However, these studies were mainly focused on women with high risk of breast cancer who were lived in urban areas [21] or only examined a limited number of risk factors [22].

Henan, Shanxi, and Sichuan locate at Eastern China, North China, and Southwest China, with GDP per capita lower than the national average level. Apart from Henan, the rest two provinces are mountainous areas, which would impede the dissemination of health service. The breast cancer incidence in Henan, Shanxi, and Sichuan province ranked in the 8^th^, 15^th^, and 32^nd^ out of 34 provinces [23], which represent the higher, middle, and lower level of breast cancer incidence in China.

Therefore, this study aimed to describe the distribution of MD and estimates the association of MD with selected known breast cancer risk factors according to menopausal status among general-risk Chinese women living in rural areas.

## Methods

### Study population

Study subjects were a subset of participants aged 45–64 years old who participated in a breast cancer screening trial that was conducted in rural areas of China. This study was led by Cancer Hospital, Chinese Academy of Medical Science and was conducted between Feb, 2018 and Feb, 2022. In this trial, women aged 35–64 years old without history of breast cancer, had lived for more than six months in their local communities, and were able to understand and provide written informed consent form (ICF) were recruited. Those who were pregnant, lactating, or plan to become pregnant at the time; had been screened for breast cancer within prior three years; had been diagnosed or treated for malignancies in the prior 12 months; showed suspicious signs of breast cancer even without indication of breast imaging exams were excluded before registration. All eligible women underwent breast ultrasound, those who aged 45–64 years old also underwent mammogram. In this study, participants aged 45–64 years old from Zezhou (Shanxi province), Xinmi (Henan Province), and Mianyang (Sichuan Province) with completed mammogram data were included.

### Mammographic density

For each participant, craniocaudal (CC) and mediolateral (MLO) views were obtained using full-field digital mammograms (SN-DR3, Shenzhen Shengnuo and Selenia Dimensions, Hologic) in each hospital and were interpreted by experienced radiologists. MD was recorded using the American College of Radiology’s Breast Imaging Reporting and Data System (BI-RADS), which categorizes the mammographic density into four categories: BI-RADS a, indicating that the breasts are almost entirely fatty; BI-RADS b, indicating that there are scattered areas of fibroglandular density; BI-RADS c, indicating that the breast are heterogeneously dense; and BI-RADS d, indicating that the breasts are extremely dense.

### Covariates

All eligible women completed the questionnaire before undertaking mammogram examination, which included demographic information (age, weight, height, education, family history of breast cancer, smoking status, and alcohol drinking status), reproductive factors (age at menarche, menopausal status, parity). Oral hormone drug use included estrogen and progesterone, and other hormone drugs such as glucocorticoids, and thyroid hormones. BMI was calculated as weight (kg) divided by height squared (m^2^) then was divided into four categories (< 18.5, 18.5–23.9, 24–27.9, 28 +) based on the criteria of weight for adults released by the National Health Commission of China in 2013. Since the number of nulliparous women was small, nulliparous women and those who have ever had one childbirth were collapsed into one group. For parous women, the cumulative time of breastfeeding was also recorded. Women who reported had no menstruation in the past 12 months were recorded as postmenopausal and age of last menstruation was recorded for them. Family history of breast cancer was defined as breast cancer occurred in first-degree, second-degree, or third-degree relatives and were grouped as binary variables because of the small number of breast cancer in each relative degree. The smoking status was defined as have never smoked (never), smoked regularly in the past six months (currently smoking), and only smoked six months before (was smoking). The alcohol drinking status was defined as have never drank (never), drank regularly in the last six months (currently drinking), only drank six months before (was drinking). The smoking and alcohol drinking status was grouped as binary variables since most of the women never smoked nor drank.

### Statistical analysis

The difference of demographic and reproductive factors among four MD categories were assessed using one-way analysis of variance (ANOVA) for continuous variable or Chi square test for categorical variables. Polytomous logistic regression was used to compare each higher MD (BI-RADS b, c, and d) to BI-RADS a, Odds Ratios (ORs) and 95% confidence intervals (95%CIs) were estimated using generalized logit model. The OR_trend_ (95%CI) and *P*_*trend*_ were estimated using cumulative logistic regression model with defining MD as an ordinal variable.

The final multivariable cumulative model included study site, age (per two years), education (none-elementary. Middle school, high school, college), BMI (per two units), age at menarche (≤ 13, 14–15, 16 +), parity (≤ 1, 2, 3 +), menopause status (pre- vs. postmenopausal), family history of breast cancer (yes vs. no). For parous women, we also adjusted age at first full term birth (≤ 20, 21–24, 25–29, 30 +) and breastfeeding (no breastfeeding, 1–6 months, 7–12 months, 13–18 months, 19–24 months, 25 +). For postmenopausal women, age at menopause (≤ 45, 45–50, 51 +) was additionally adjusted. Smoking and oral hormone drug use were not associated with MD in the univariate analysis and therefore was not included in the final multivariable analysis. We also excluded the alcohol drinking from final analysis because only 347 (2.77%) women drank alcohol currently or six months before. We also did stratified analysis with menopausal status as the stratified factors. The MD was grouped as dense group (BI-RADS c-d) and non-dense group (BI-RADS a-b) in stratified analysis due to the small number of participants in some extreme groups. The significance level was 0.05 for two-sided tests. All statistical analysis was performed using SAS 9.4 software (SAS Institute Inc., Cary, NC).

## Results

### Baseline characteristics of the study participants

A total of 12,518 women aged 45–64 years were included in this analysis. The demographic characteristics were described in Table 1. Overall, the mean age was 51.53 (SD, 4.45) years and the mean BMI was 23.9 kg/m^2^ (SD: 2.26). 50.16% of the participants were postmenopausal. 39.66% of the women had more than nine years of education (high school and above). Most women were parous (99.24%), and reported that they gave first full term birth at age 21–29 (92.20%), had breastfed (95.88%). Almost all participants reported that they had never smoked (99.59%) nor drank alcohol (97.23). 1.66% of the women reported a family history of breast cancer within three blood degrees.

**Table 1:** Demographic characteristics and breast cancer risk factors of participants

	**All**			**BI-RADS a**		**BI-RADS b**		**BI-RADS c**		**BI-RADS d**		***P***^a^
	***n* = 12,518**			***n* = 693**		***n* = 3042**		***n* = 6717**		***n* = 2066**		
	**n**	**%**		**n**	**%**	**n**	**%**	**n**	**%**	**n**	**%**	
**age**												
**Mean (SD)**	51.53	4.45		55.28	4.53	53.46	4.58	51.04	4.11	49.00	3.15	< 0.01
**45–49**	5394	43.09		102	14.72	769	25.28	3107	46.26	1416	68.54	< 0.01
**50–54**	4237	33.85		204	29.44	1110	36.49	2385	35.51	538	26.04	
**55–59**	2303	18.40		275	39.68	888	29.19	1037	15.44	103	4.99	
**60–64**	584	4.67		112	16.16	275	9.04	188	2.80	9	0.44	
**education**												
**None-elementary**	1987	15.88		144	20.81	532	17.49	1103	16.43	208	10.07	< 0.01
**Middle school**	5563	44.46		354	51.16	1436	47.22	2951	43.95	822	39.79	
**High school**	2547	20.35		124	17.92	680	22.36	1328	19.78	415	20.09	
**College and above**	2416	19.31		70	10.12	393	12.92	1332	19.84	621	30.06	
**missing**	5			1		1		3		0		
**BMI (kg/m**^**2)**^												
**Mean (SD)**	23.9	2.66		25.36	2.91	24.38	2.72	23.85	2.58	22.85	2.30	< 0.01
** < 18.5**	120	0.96		2	0.29	23	0.76	55	0.82	40	1.94	< 0.01
**18.5–23.9**	6644	53.12		235	33.96	1393	45.85	3594	53.55	1422	68.86	
**24–27.9**	4837	38.67		332	47.98	1323	43.55	2622	39.06	560	27.12	
**28 + **	906	7.24		123	17.77	299	9.84	441	6.57	43	2.08	
**missing**	11			1		4		5		1		
**age at menarche**												
** ≤ 13**	3837	30.66		189	27.27	859	28.26	2141	31.87	648	31.40	< 0.01
**14–15**	4824	38.55		227	32.76	1125	37.01	2597	38.66	875	42.39	
**16 + **	3853	30.79		277	39.97	1056	34.74	1979	29.46	541	26.21	
**missing**	4			0		2		0		2		
**parity**												
**0**	95	0.76		3	0.43	17	0.56	51	0.76	24	1.16	< 0.01
**1**	4501	36.00		119	17.17	929	30.60	2504	37.30	949	46.05	
**2**	6383	51.05		394	56.85	1594	52.50	3428	51.07	967	46.92	
**3 + **	1524	12.19		177	25.54	496	16.34	730	10.87	121	5.87	
**missing**	15			0		6		4		5		
**age at first full term birth**^b^												
** ≤ 20**	537	4.33		22	3.19	138	4.57	307	4.61	70	3.44	< 0.01
**21–24**	7133	57.52		414	60.00	1840	60.97	3777	56.75	1102	54.13	
**25–29**	4300	34.68		231	33.48	964	31.94	2324	34.92	781	38.36	
**30 + **	430	3.47		23	3.33	76	2.52	248	3.73	83	4.08	
**missing**	8			0		1		6		1		
**breastfeeding**^b^												
**No breastfeeding**	510	4.12		18	2.61	99	3.29	298	4.49	95	4.67	< 0.01
**1–6 months**	661	5.34		12	1.74	163	5.41	373	5.61	113	5.56	
**7–12 months**	3169	25.60		107	15.51	686	22.78	1694	25.50	682	33.55	
**13–18 months**	1420	11.47		64	9.28	320	10.63	766	11.53	270	13.28	
**19–24 months**	2993	24.18		195	28.26	769	25.54	1541	23.19	488	24.00	
**25 months + **	3625	29.29		294	42.61	974	32.35	1972	29.68	385	18.94	
**missing**	30			0		8		18		4		
**menopausal status**												
**premenopausal**	6228	49.84		113	16.31	879	28.95	3626	54.06	1610	78.12	< 0.01
**postmenopausal**	6269	50.16		580	83.69	2157	71.05	3081	45.94	451	21.88	
**missing**	21			0		6		10		5		
**age at menopause**^c^												
** ≤ 45**	1009	16.13		116	20.03	329	15.30	474	15.40	90	20.04	< 0.01
**46–50**	3208	51.28		271	46.80	1111	51.65	1593	51.77	233	51.89	
**51 + **	2039	32.59		192	33.16	711	33.05	1010	32.82	126	28.06	
**missing**	13			1		6		4		2		
**oral hormone drug use**												
**never**	12,229	98.61		675	98.54	2947	98.10	6582	98.75	2025	98.93	0.12
**1–5 years**	134	1.08		9	1.31	44	1.46	66	0.99	15	0.73	
**5 years + **	38	0.31		1	0.15	13	0.43	17	0.26	7	0.34	
**missing**	117			8		38		52		19		
**family history of breast cancer**												
**no**	12,289	98.31		688	99.28	2985	98.42	6602	98.39	2014	97.58	< 0.05
**yes**	211	1.69		5	0.72	48	1.58	108	1.61	50	2.42	
**missing**	18			0		9		7		2		
**smoking**												
**never**	12,452	99.50		693	1.00	3022	99.44	6680	99.46	2057	99.56	0.25
**yes**	62	0.50	0	0	0.00	17	0.56	36	0.54	9	0.44	
**missing**	4			0		3		1		0		
**alcohol drinking**												
**never**	12,167	97.23		688	99.28	2933	96.51	6540	97.38	2006	97.10	< 0.01
**yes**	347	2.77	0	5	0.72	106	3.49	176	2.62	60	2.90	
**missing**	4			0		3		1		0		

^a^results are from one-way analysis of variance (ANOVA) for continuous variables and Chi square test for categorical variables

^b^parous women only

^c^postmenopausal women only

### The distribution of mammographic density

**Fig. 1:**
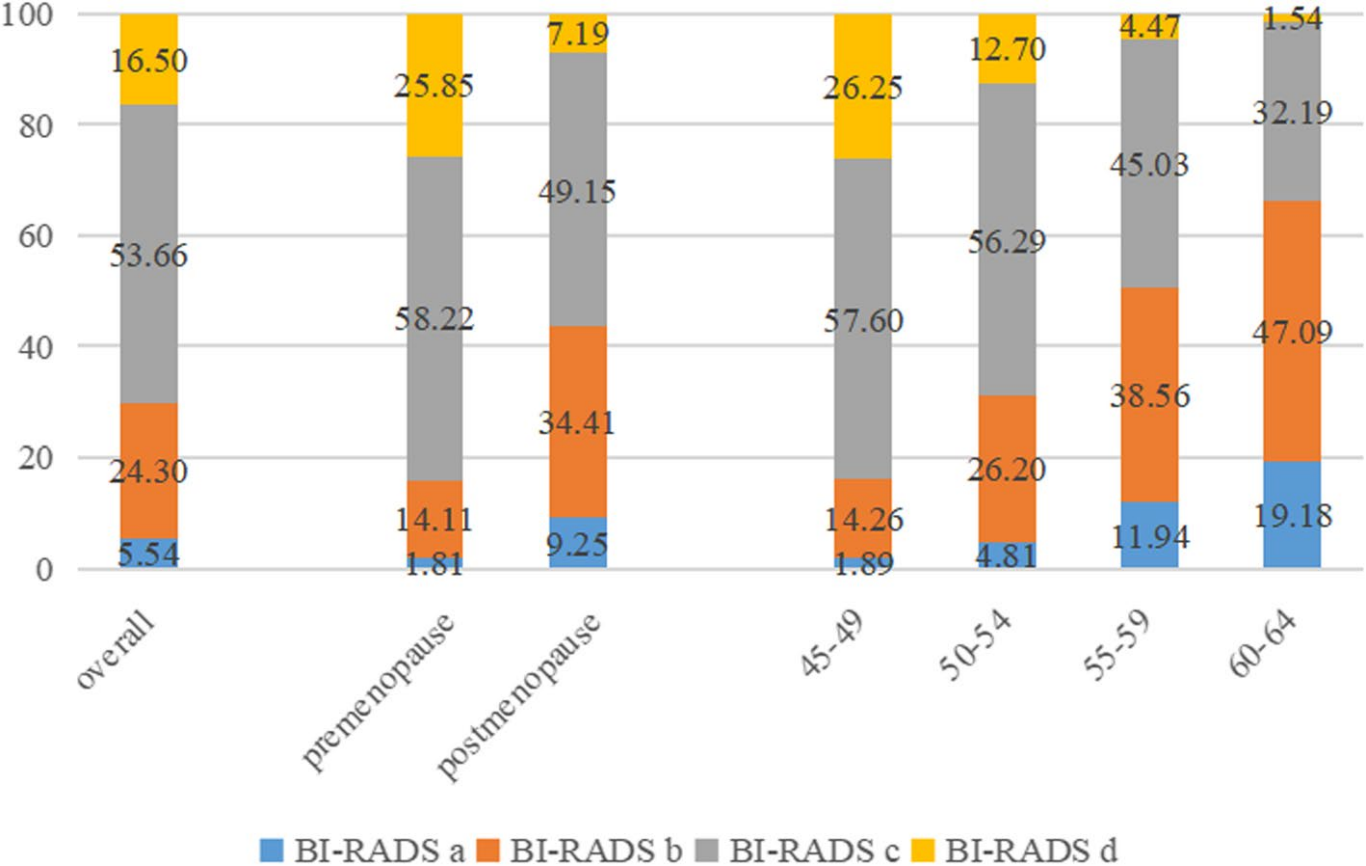
Distribution of MD by age groups and menopausal status

**Fig. 2:**
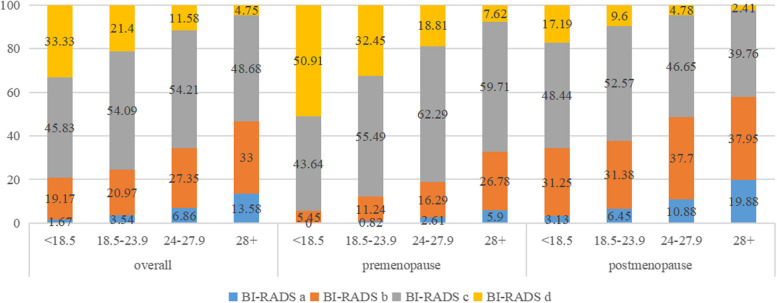
Distribution of MD by BMI stratified by menopausal status

The distribution of mammographic density by age groups and menopausal status were shown in Fig. 1. Overall, the proportion of mammographic density rated as almost entirely fatty (BI-RADS a), scattered fibrograndular densities (BI-RADS b), heterogeneously dense (BI-RADS c), and extremely dense (BI-RADS d) were 5.54%, 24.30%, 53.66%, and 16.50%, respectively. The proportion of dense breasts (BI-RADS c-d) decreased from 83.85% for women aged 45–49 to 33.73% for women aged 60–64 (*P*_*trend*_ < 0.01). MD also decreased with BMI both in premenopausal and postmenopausal women (*P*_*trend*_ < 0.01) (Fig. 2). MD also varied by level of education, age at menarche, parity, age at first full term birth, breastfeeding, menopause status, family history of breast cancer, alcohol drinking (*P *_*Chi-square or ANOVA*_ < 0.05, Table 1).

### Factors associated with MD

The association between selected demographic and reproductive factors with MD was examined using multivariable polytomous regression model and cumulative logistic regression model. We found than older age (OR_trend_ = 0.81, 95%CI: 0.79–0.81, per 2-year increase), higher BMI (OR_trend_ = 0.73, 95%CI: 0.71–0.75, per 2 kg/m^2^), more births (OR_trend_ = 0.47, 95%CI: 0.41–0.54, 3 + vs. 0–1), postmenopausal status (OR_trend_ = 0.42, 95%CI: 0.0.38–0.46) were associated with lower MD (Table 2). For parous women, longer duration of breastfeeding was found to be associated with higher MD when adjusting for study site age, BMI, and age of first full-term birth (OR_trend_ = 1.53, 95%CI: 1.27–1.85, 25 + months vs. no breastfeeding; OR_trend_ = 1.45, 95%CI: 1.20–1.75, 19–24 months vs. no breastfeeding), however, the association became non-significant when adjusting all covariates (Table 2). Associations between examined risk factors and MD were similar in premenopausal and postmenopausal women except for level of education and oral hormone drug usage. Higher education was only found to be associated with an increased proportion of dense breasts in postmenopausal women (OR_trend_ = 1.08, 95%CI: 1.02–1.15). Premenopausal women who ever used oral hormone drug were less likely to have dense breasts, though the difference was marginally significant (OR = 0.54, *P* = 0.045). We observed that BMI had stronger effect on reducing MD among premenopausal women than postmenopausal women (OR_pre_ = 0.70, 95%CI: 0.66–0.74; OR_post_ = 0.78, 95%CI: 0.75–0.81). In postmenopausal women, we also found the proportion of dense breasts increased with age at menopause (OR_trend_ = 1.31, 95%CI: 1.21–1.43) (Table 3).

**Table 2:** Associations between selected characteristics and mammographic density

	**BI-RADS b vs. a**	**BI-RADS c vs. a**	**BI-RADS d vs. a**	**OR**_**trend**_** (95%CI)**^b^	***P***_***trend***_^b^
	**OR (95%CI)**^a^	**OR (95%CI)**^a^	**OR (95%CI)**^a^		
**age**					
**per 2 years**	0.88 (0.84–0.92)	0.75 (0.71–0.78)	0.62 (0.59–0.66)	0.81 (0.79–0.82)	< 0.01
**education**					
**None-elementary**	1	1	1	1	
**Middle school**	1.07 (0.85–1.34)	0.96 (0.77–1.20)	0.99 (0.75–1.29)	0.91 (0.83–1.01)	0.08
**High school**	1.42 (1.07–1.89)	1.27 (0.97–1.68)	1.46 (1.05–2.03)	1.00 (0.88–1.12)	0.93
**College**	0.87 (0.61–1.24)	1.00 (0.70–1.41)	1.22 (0.83–1.80)	1.14 (1.00–1.30)	< 0.05
**BMI**					
**per 2 kg/m**^**2**^	0.78 (0.74–0.83)	0.64 (0.60–0.68)	0.46 (0.43–0.50)	0.73 (0.71–0.75)	< 0.01
**age at menarche**					
** ≤ 13**	1	1	1	1	
**14–15**	1.25 (1.00–1.56)	1.21 (0.98–1.51)	1.22 (0.96–1.55)	1.04 (0.95–1.13)	0.41
**16 + **	1.16 (0.93–1.44)	1.08 (0.87–1.34)	1.07 (0.84–1.37)	0.98 (0.90–1.08)	0.70
**parity**					
** ≤ 1**	1	1	1	1	
**2**	0.89 (0.69–1.16)	0.61 (0.48–0.79)	0.47 (0.36–0.61)	0.65 (0.59–0.71)	< 0.01
**3 + **	0.72 (0.53–0.98)	0.37 (0.27–0.50)	0.24 (0.17–0.35)	0.47 (0.41–0.54)	< 0.01
**age at first full term birth**^c^					
** ≤ 20**	1	1	1	1	
**21–24**	0.83 (0.51–1.34)	0.81 (0.50–1.30)	0.83 (0.49–1.42)	0.96 (0.81–1.14)	0.64
**25–29**	0.73 (0.44–1.19)	0.76 (0.47–1.25)	0.74 (0.43–1.29)	0.94 (0.78–1.13)	0.49
**30 + **	0.38 (0.19–0.75)	0.45 (0.23–0.88)	0.45 (0.21–0.94)	0.86 (0.66–1.11)	0.25
**breastfeeding**^c^					
**No breastfeeding**	1	1	1		
**1–6 months**	1.16 (0.52–2.60)	0.89 (0.40–1.97)	1.10 (0.47–2.56)	0.94 (0.74–1.18)	0.59
**7–12 months**	1.00 (0.56–1.77)	0.82 (0.47–1.43)	0.89 (0.49–1.61)	0.95 (0.79–1.14)	0.58
**13–18 months**	0.99 (0.55–1.80)	0.76 (0.42–1.35)	0.76 (0.41–1.43)	0.87 (0.71–1.06)	0.16
**19–24 months**	0.99 (0.56–1.75)	0.78 (0.45–1.36)	0.72 (0.39–1.31)	0.83 (0.68–1.01)	0.06
**25 months + **	1.02 (0.58–1.80)	0.91 (0.52–1.58)	0.75 (0.41–1.37)	0.89 (0.73–1.09)	0.25
**menopausal status**					
**premenopausal**	1	1	1	1	
**postmenopausal**	0.60 (0.46–0.77)	0.29 (0.23–0.38)	0.16 (0.12–0.21)	0.42 (0.38–0.46)	< 0.01
**age at menopause**^d^					
** ≤ 45**	1	1	1	1	
**46–50**	1.52 (1.17–1.97)	1.75 (1.35–2.27)	1.70 (1.18–2.44)	1.25 (1.09–1.44)	< 0.01
**51 + **	1.78 (1.34–2.36)	2.66 (2.00–3.53)	3.46 (2.26–5.28)	1.70 (1.45–1.98)	< 0.01
**family history of breast cancer**					
**no**	1	1	1	1	
**yes**	1.92 (0.75–4.93)	1.77 (0.69–4.51)	2.34 (0.88–6.22)	1.18 (0.90–1.54)	0.23

^a^Polytomous logistic regression was used to estimate ORs and 95%CIs comparing each BI-RADS categories of (b, c, d) to BI-RADS a. adjusted factors: study site, age, education, BMI, age at menarche, parity, menopausal status, family history of breast cancer

^b^Cumulative logistic regression and Wald Chi square test was used to estimate OR_trend_ and *P*_*trend*_ with MD modeled as an ordinal variable comparing higher to lower BI-RADS categories. Model was adjusted for the same factors as above

^c^Parous women only. Model was additionally adjusted for age at first full term birth and breastfeeding

^d^postmenopausal women only

**Table 3:** Associations between selected characteristics and mammographic density by menopausal status

	**Premenopausal (*n* = 6228)**		**Postmenopausal (*n* = 6269)**	
	**OR (95%CI)**^a, b^	***P***	**OR (95%CI)**^a, c^	***P***
**age**				
**per 2 years**	0.83 (0.78–0.87)	< 0.01	0.81 (0.79–0.83)	< 0.01
**education**				
**None-elementary**	1		1	
**Middle school**	0.99 (0.79–1.24)	0.94	0.86 (0.74–1.00)	0.05
**High school**	0.92 (0.71–1.20)	0.54	0.98 (0.82–1.16)	0.79
**College**	1.10 (0.83–1.44)	0.52	1.25 (1.01–1.55)	0.04
**OR**_**trend**_** (95%CI); *P***_***trend***_^d^	1.03 (0.95–1.12)	0.47	1.08 (1.02–1.15)	< 0.05
**BMI**				
**per 2 kg/m**^**2**^	0.70 (0.66–0.74)	< 0.01	0.78 (0.75–0.81)	< 0.01
**age at menarche**				
** ≤ 13**	1		1	
**14–15**	0.94 (0.79–1.11)	0.48	1.06 (0.92–1.21)	0.43
**16 + **	0.91 (0.75–1.10)	0.33	0.99 (0.86–1.14)	0.90
**OR**_**trend**_** (95%CI); *P***_***trend***_^d^	0.99 (0.90–1.08)	0.76	1.02 (0.96–1.10)	0.51
**parity**				
**0–1**	1		1	
**2**	0.62 (0.51–0.75)	< 0.01	0.64 (0.56–0.75)	< 0.01
**3 + **	0.49 (0.36–0.65)	< 0.01	0.44 (0.36–0.54)	< 0.01
**OR**_**trend**_** (95%CI); *P***_***trend***_^d^	0.82 (0.72–0.93)	< 0.01	0.80 (0.73–0.87)	< 0.01
**age at first full term birth**^e^				
** ≤ 20**	1		1	
**21–24**	0.96 (0.69–1.32)	0.79	0.97 (0.72–1.30)	0.83
**25–29**	0.89 (0.63–1.25)	0.49	1.08 (0.80–1.47)	0.61
**30 + **	1.25 (0.74–2.09)	0.41	0.89 (0.57–1.38)	0.60
**OR**_**trend**_** (95%CI); *P***_***trend***_^d^	1.01 (0.89–1.14)	0.91	1.12 (1.02–1.23)	< 0.05
**breastfeeding**^e^				
**No breastfeeding**	1		1	
**1–6 months**	1.05 (0.63–1.75)	0.85	0.69 (0.47–1.01)	0.05
**7–12 months**	0.94 (0.63–1.40)	0.76	0.80 (0.58–1.08)	0.15
**13–18 months**	0.82 (0.54–1.27)	0.38	0.73 (0.53–1.02)	0.07
**19–24 months**	0.88 (0.58–1.34)	0.55	0.72 (0.52–1.00)	0.05
**25 months + **	0.94 (0.61–1.44)	0.78	0.83 (0.60–1.15)	0.26
**OR**_**trend**_** (95%CI); *P***_***trend***_^d^	1.03 (0.97–1.10)	0.33	1.06 (1.01–1.11)	< 0.05
**age at menopause**				
** ≤ 45**	-	-	1	
**46–50**	-	-	1.23 (1.06–1.44)	< 0.01
**51 + **	-	-	1.70 (1.43–2.02)	< 0.01
**OR**_**trend**_** (95%CI); *P***_***trend***_^d^	-	-	1.31 (1.21–1.43)	< 0.01
**oral hormone drug use**				
**never**	1		1	
**yes**	0.54 (0.30–0.99)	0.045	0.85 (0.58–1.27)	0.43
**family history of breast cancer**				
**no**	1		1	
**yes**	1.05 (0.61–1.81)	0.87	1.07 (0.68–1.68)	0.78

^a^Binary logistic regression was used to estimate the ORs and 95%CIs comparing the dense (BI-RADS a-b) to non-dense breast group (BI-RADS c-d)

^b^Adjusted for study site, age, education, BMI, age at menarche, parity, family history of breast cancer

^c^Adjusted for study site, age, education, BMI, age at menarche, parity, family history of breast cancer, age at menopause

^d^OR_trend_ and *P*_*trend*_ were estimated and obtained by treating categorical variables as continuous variables

^e^Parous women only

**Fig. 3:**
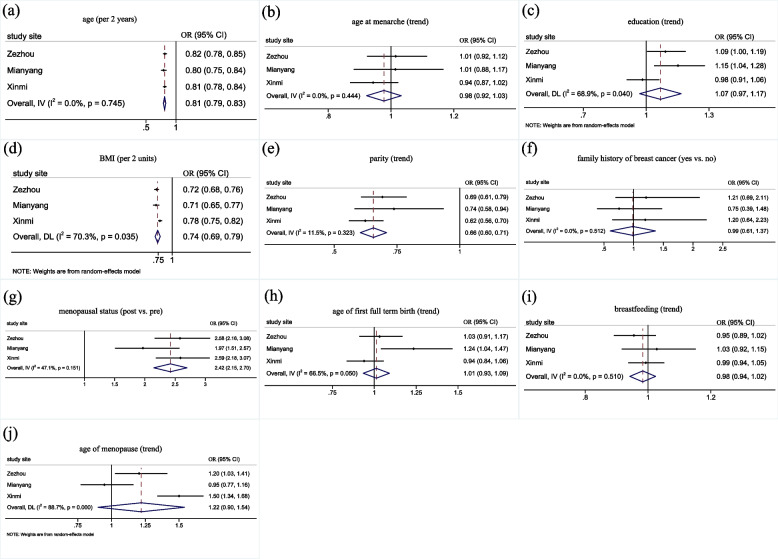
Associations between risk factors with MD by study site: **a** age, **b** age at menarche, **c** education, **d** BMI, **e** parity, **f** family history of breast cancer, **g** menopausal status, **h** age of first full term birth, **i** breastfeeding, **j** age of menopause. The study site specific OR_trend_ for having dense breasts (BI-RADS c-d) compared with non-dense breasts (BI-RADS a-b) were estimated by treating categorical variables as continuous variables. All models were adjusted for age (per 2 years), BMI (per 2 units), education (none-elementary, middle school, high school, college), age at menarche (≤ 13, 14–15, 16 +), parity (≤ 1, 2, 3 +), menopause status (pre vs. post), family history of breast cancer (yes vs. no). Age of first full term birth (≤ 20, 21–24, 25–29, 30 +) and breastfeeding (no breastfeeding, 1–6 months, 7–12 months, 13–18 months, 19–24 months, 25 months +) was estimated only for parous women and adjusted by each other. The age of menopause (≤ 45, 46–50, 51 +) was estimated only for postmenopausal women

Associations of MD with examined risk factors were assessed by study sites using meta-analysis (Fig. 3). Among all risk factors, the significant heterogeneity for education, BMI, age at menopause (*P*_*heterogeneity*_ < 0.05) were found among three study sites. The demographic characteristics of participants from different study sites were available in Additional file Table S1. In sensitivity analysis, we analyzed the association of the same factors when combining MD into two categories (dense breasts vs. non-dense breasts) and observed the same associations (data are available in Additional file Table S2).

## Discussion

In this study, we described the distribution of MD among Chinese women with average risk for breast cancer and assessed the associations between known breast cancer risk factors with MD in this population. To the best of our knowledge, this is the first large study conducted among women with general risk of breast cancer in rural China. The associations we found in this study were consistent with that reported in previous studies, and were similar in pre-menopausal and postmenopausal women.

Previous studies comparing the MD between Caucasian and Asian women found that Asian women had higher percent density and dense volume, especially in premenopausal woman [17, 19]. In our study, approximately 60% of the women aged 45–64 years were classified as heterogeneously dense or extremely dense breasts, this number was even higher in women aged 45–49 years. Our result was higher than Sung et al. [21] (55%, age: 45–69 years) and Dai et al. [22] (49%, age: 45–64 years) had reported which conducted among urban women. This could be explained by differences in life and diet styles [14] between those living in urban and rural areas. Higher MD does not necessarily lead to higher incidence of breast cancer due to the complexity etiology of breast cancer including age, family history of breast cancer [2], age of menarche, age of first full-term pregnancy, HRT use [3], genetic mutation [4], BMI [5], alcohol consumption [6], and physical inactivity [7]. The older demographics, fewer childbirth, and older age at first full-term pregnancy of the urban women may contribute to the higher incidence of breast cancer in this population.

The high proportion of dense breasts in rural women highlighted that accessible and accurate breast cancer screening modalities other than mammography are needed for those women living in rural areas since they are more likely to have dense breasts. Previous studies found an increased sensitivity of adding digital breast tomosynthesis (DBT), hand-held ultrasound, automated breast ultrasound, and magnetic resonance imaging (MRI) to MG in women with either higher risk of breast cancer or dense breasts [24]. To maximize benefits using limited resources, which is the problems of rural areas, adopting ultrasound-based breast cancer screening in rural areas might be a feasible approach. On the other hand, developing a risk-stratified screening program incorporating MD with other common risk factors could improve resource allocation [25].

Age and BMI, as expected, were inversely associated with MD. Previous studies found that MD decreased with age in healthy Taiwanese women and Western women [26]. Breast lobules involution that characterized by the reduction in number and size of acini in lobules is positively associated with increasing aging. Furthermore, breast glandular elements and collagen are progressively replaced by fatty tissue as women aging [27]. These two mechanisms might explain the inverse association between age and MD. However, lobular involution and fatty areas were found to be association with reduced risk of breast cancer [27–29]. Cumulative exposure to MD, which reflects the cumulative exposure to factors that promote the carcinogenesis, may explain the paradox observation that with increasing age, MD decreases and breast lobular regresses, whereas breast cancer risk increases [8, 10, 30, 31]. BMI is positively associated with non-dense area of the mammogram, thus, is negatively associated with MD [32]. BMI and MD are positively associated with increased breast cancer risk in postmenopausal women, but are inversely associated with each other. Norman F. Body et al. noted that BMI and MD could independently perform through different pathways in breast cancer development [33]. In our study, we found BMI had stronger effect on reducing MD in premenopausal women. Norman F. Boyd et.al. also found a stronger inverse association between BMI and MD in both premenopausal breast cancer patients and non-patients than postmenopausal women.

The association between parity and MD was well-established through previous studies. Parous status and higher number of births were associated with decreased MD in Chinese and other populations [21, 22, 34]. Menopausal status was also proven to be independently associated with MD from prior studies [21, 22, 34, 35]. Our findings showed an inverse association between number of births, menopausal status and MD after adjustment for age and other cofounders, which were in consistent with previous evidences. In our study, longer than 19 months of breastfeeding was found to be significantly associated with decreased MD when adjusting for study site, age, BMI, and age of first full-term birth though the association became nonsignificant when additionally adjusted for education, age at menarche, menopausal status and family history of breast cancer. Advanced age at first full term birth and limited breastfeeding were associated with increased risk of breast cancer both in China and other countries [3, 36]. However, their associations with MD are less consistent and more investigations are warranted [34, 37–39].

Previous studies evaluating relationships between age at menarche and MD produced inconsistent results. Most reported finding no evidence of an association between age at menarche and MD, some reported a positive association between them, while very few reported an inverse association [34]. Sarah V. Ward et al. first demonstrated associations between age at menarche and MD across 22 different countries. They found a small positive association between later age at menarche and both increasing per cent and absolute dense area [40]. Both Dai, Hongji et al. [22] and Hyuna Sung et al. [21] found no evidence of an association between age at menarche and MD in Chinese populations, which is consistent with our result. Amita G. Ghadge et al. [41] reported that pubertal mammary gland development may affect adult MD and cancer risk through complexed mechanisms involving endocrine regulators, paracrine regulator, genetic and epigenetic determinants. Therefore, the paradox epidemiological findings may be attributed to the complex mechanisms of mammary gland development.

Estrogen and progesterone, which are the main components of hormone replacement therapy, are important in the development of breast cancer, especially hormone receptor-related tumors [42]. Valerie A.M. al. reported that MD increased by 2.4% with the use of hormone therapy [43]. Celia Byrne et al. investigated the effect of estrogen and progestin therapy on MD by randomly assigning participants to estrogen plus progestin or placebo therapy, they found that mean MD increased by 9.73% after at least one year of using estrogen and progestin therapy and this change was associated with increased breast cancer risk [44], Norman F. Boyd et al. reported that the association between MD change and hormone therapy in postmenopausal women were greater in women who later developed breast cancer than those who didn’t [45]. However, another study assessed the association between blood levels of estradiol, progesterone, prolactin, sex hormone binding globulin and MD change found that total estradiol and progesterone levels were unrelated to MD in both pre- and post- menopausal women [46]. In our study, a small number of women who ever used hormone drug. We found only marginal evidence that oral hormone drug use in premenopausal women were less likely to have dense breasts. In our study, in addition to progesterone and estrogen, glucocorticoids and thyroid hormones were also recorded as hormone drug use, which accounted for 9.5% (8/84) of premenopausal women whoever took hormone drugs. This might confound the association between sex hormone and MD. Apart from epidemiological evidences, there is a need for more researches on the underlying mechanisms between hormone factors and MD, and the genetic and environmental factors that influence the levels of these factors.

The main strength of this study was the large number of understudied Chinese women that came from areas with limited medical resources. We also evaluated associations for pre- and pos- tmenopausal women separately. Limitations of this study include the visual and qualitative assessment of MD, which is user dependent. Quantitative assessments convey additional information on breast density. However, Inger T Gram et al. reported that both the qualitative and quantitative methods capture the same overall associations with risk factors for breast cancer in postmenopausal women [47]. Computer-assisted methods are highly consistent and producible, but have limited application because they require either digital mammography or trained observer, whereas BI-RADS score is widely used in routine breast cancer screening activities. Future studies using computer-assisted methods could provide more accurate estimation of MD. All information about potential breast cancer risk factors were self-reported, thus the accuracy may be suboptimal. Questionnaires were checked for any logical error or missing value by project team members before each participant leaving the study sites, minimized the error rates.

In conclusion, most of the Chinese women with general risk for breast cancer have dense breasts. We found MD was associated with some established breast cancer risk factors. This finding may help to understand the etiology of breast cancer and have implications for breast cancer prevention in low resource areas where mammographic screening is not practicable to general population.

### Supplementary Information

**Additional file 1: Table S1** Demographic characteristics and breast cancer risk factors of participants and **Table S2** Associations between selected characteristics and mammographic density are included in the additional file. **Table S1.** Described the demographic characteristics and breast cancer risk factors of participants from each study sites. **Table S2.** Showed results of association of the same factors when combining MD into two categories (dense breasts vs. non-dense breasts).

## Data Availability

The datasets used and/or analyzed during the current study are available from the corresponding author on reasonable request.

## Abbreviations

MD: Mammographic density
BI-RADS: Breast imaging reporting and data system
OR: Odds ratio
CI: Confidence interval
BMI: Body mass index
ICF: Informed consent form
CC: Craniocaudal
MLO: Mediolateral
ANOVA: One-way analysis of variance
