# Supplementary material for: Breast cancer risk factors and mammographic density among 12518 average-risk women in rural China
Source: BMC Cancer. 2023 Oct 9;23:952. doi: 10.1186/s12885-023-11444-7 (PMC10561452; doi:10.1186/s12885-023-11444-7)
Supplement: Supplementary file 1 — Additional file 1: Table S1 Demographic characteristics and breast cancer risk factors of participants and Table S2 Associations between selected characteristics and mammographic density are included in the additional file. Table S1. Described the demographic characteristics and breast cancer risk factors of participants from each study sites. Table S2. Showed results of association of the same factors when combining MD into two categories (dense breasts vs. non-dense breasts). [file 12885_2023_11444_MOESM1_ESM.docx]

**Additional file**

**Breast cancer risk factors and mammographic density among 12518 average-risk women in rural China**

Huijiao Yan1, Wenhui Ren2, Mengmeng Jia1, Peng Xue1, Zhifang Li3, Shaokai Zhang4, Lichun He5, Youlin Qiao1#

1 School of Population Medicine and Public Health, Chinese Academy of Medical Sciences and Peking Union Medical College, Beijing, 100730, China

2 National Cancer Center/National Clinical Research Center for Cancer/Cancer Hospital, Chinese Academy of Medical Sciences and Peking Union Medical College, Beijing, 100021, China

3 Changzhi Medical College, Changzhi 046000, Shanxi, China

4 Department of Cancer Epidemiology, The Affiliated Cancer Hospital of Zhengzhou University & Henan Cancer Hospital, Henan Engineering Research Center of Cancer Prevention and Control, Henan International Joint Laboratory of Cancer Prevention, Zhengzhou, 450008, China

5 Mianyang Maternal & Child Health Care Hospital, Mianyang Children’s Hospital, Mianyang, 621000, China

| **Table S1. Demographic characteristics and breast cancer risk factors of participants** | | | | | | | | |
| --- | --- | --- | --- | --- | --- | --- | --- | --- |
|  | **All** | | **Zezhou** | | **Mianyang** | | **Xinmi** | |
|  | **n=12518** | | **n=4529** | | **n=2064** | | **n=5925** | |
|  | **n** | **%** | **n** | **%** | **n** | **%** | **n** | **%** |
| **Age** |  |  |  |  |  |  |  |  |
| **Mean (SD)** | 51.53 | 4.45 | 50.89 | 4.44 | 51.19 | 4.48 | 52.14 | 4.37 |
| **45-49** | 5394 | 43.09 | 2258 | 49.86 | 979 | 47.43 | 2157 | 36.41 |
| **50-54** | 4237 | 33.85 | 1389 | 30.67 | 658 | 31.88 | 2190 | 36.96 |
| **55-59** | 2303 | 18.40 | 694 | 15.32 | 323 | 15.65 | 1286 | 21.70 |
| **60-64** | 584 | 4.67 | 188 | 4.15 | 104 | 5.04 | 292 | 4.93 |
| **Education** |  |  |  |  |  |  |  |  |
| **None-elementary** | 1987 | 15.88 | 332 | 20.81 | 334 | 17.49 | 1321 | 16.43 |
| **Middle school** | 5563 | 44.46 | 2113 | 46.65 | 598 | 29.02 | 2852 | 48.15 |
| **High school** | 2547 | 20.35 | 948 | 20.93 | 440 | 21.35 | 1159 | 19.57 |
| **College** | 2416 | 19.31 | 1136 | 25.08 | 689 | 33.43 | 591 | 9.98 |
| **missing** | 5 |  | 0 |  | 3 |  | 2 |  |
| **BMI (kg/m^2)^** |  |  |  |  |  |  |  |  |
| **Mean (SD)** | 23.9 | 2.66 | 23.48 | 2.40 | 23.09 | 2.69 | 24.49 | 2.70 |
| **<18.5** | 120 | 0.96 | 41 | 0.91 | 41 | 2.00 | 38 | 0.64 |
| **18.5-23.9** | 6644 | 53.12 | 2730 | 60.28 | 1334 | 64.91 | 2580 | 43.56 |
| **24-27.9** | 4837 | 38.67 | 1577 | 34.82 | 569 | 27.69 | 2691 | 45.43 |
| **28+** | 906 | 7.24 | 181 | 4.00 | 111 | 5.40 | 614 | 10.37 |
| **missing** | 11 |  |  |  | 9 |  | 2 |  |
| **Age at menarche** |  |  |  |  |  |  |  |  |
| **≤13** | 3837 | 30.66 | 1147 | 25.33 | 952 | 46.21 | 1738 | 29.33 |
| **14-15** | 4824 | 38.55 | 1908 | 42.13 | 740 | 35.92 | 2176 | 36.73 |
| **16+** | 3853 | 30.79 | 1474 | 32.55 | 368 | 17.86 | 2011 | 33.94 |
| **missing** | 4 |  | 0 |  | 4 |  | 0 |  |
| **parity** |  |  |  |  |  |  |  |  |
| **≤1** | 4596 | 36.76 | 1719 | 37.96 | 1732 | 84.49 | 1145 | 19.33 |
| **2** | 6383 | 51.05 | 2480 | 54.76 | 272 | 13.27 | 3631 | 61.29 |
| **3+** | 1524 | 12.19 | 330 | 7.29 | 46 | 2.24 | 1148 | 19.38 |
| **missing** | 15 |  | 0 |  | 14 |  | 1 |  |
| **age at first full term birth ^a^** |  |  |  |  |  |  |  |  |
| **≤20** | 537 | 4.33 | 106 | 2.36 | 138 | 6.90 | 293 | 4.97 |
| **21-24** | 7133 | 57.52 | 2519 | 55.98 | 877 | 43.85 | 3737 | 63.34 |
| **25-29** | 4300 | 34.68 | 1719 | 38.20 | 829 | 41.45 | 1752 | 29.69 |
| **30+** | 430 | 3.47 | 156 | 3.47 | 156 | 7.80 | 118 | 2.00 |
| **missing** | 8 |  | 1 |  | 3 |  | 4 |  |
| **Breastfeeding ^a^** |  |  |  |  |  |  |  |  |
| **No breastfeeding** | 510 | 4.12 | 148 | 3.29 | 149 | 7.46 | 213 | 3.62 |
| **1-6 months** | 661 | 5.34 | 105 | 2.33 | 456 | 22.83 | 100 | 1.70 |
| **7-12 months** | 3169 | 25.60 | 1429 | 31.76 | 1010 | 50.58 | 730 | 12.41 |
| **13-18 months** | 1420 | 11.47 | 513 | 11.40 | 186 | 9.31 | 721 | 12.26 |
| **19-24 months** | 2993 | 24.18 | 1474 | 32.76 | 113 | 5.66 | 1406 | 23.91 |
| **25 months+** | 3625 | 29.29 | 831 | 18.47 | 83 | 4.16 | 2711 | 46.10 |
| **missing** | 30 |  | 1 |  | 6 |  | 23 |  |
| **menopausal status** |  |  |  |  |  |  |  |  |
| **premenopausal** | 6228 | 49.84 | 2720 | 60.06 | 902 | 43.79 | 2606 | 44.11 |
| **postmenopausal** | 6269 | 50.16 | 1809 | 39.94 | 1158 | 56.21 | 3302 | 55.89 |
| **missing** | 21 |  | 0 |  | 4 |  | 17 |  |
| **age at menopause ^b^** |  |  |  |  |  |  |  |  |
| **≤45** | 1009 | 16.13 | 271 | 5.98 | 203 | 17.58 | 535 | 16.25 |
| **46-50** | 3208 | 51.28 | 922 | 20.36 | 666 | 57.66 | 1620 | 49.21 |
| **51+** | 2039 | 32.59 | 616 | 13.60 | 286 | 24.76 | 1137 | 34.54 |
| **missing** | 13 |  | 0 |  | 3 |  | 10 |  |
| **oral hormone drug use** |  |  |  |  |  |  |  |  |
| **never** | 12229 | 98.61 | 4448 | 98.52 | 1968 | 97.19 | 5813 | 99.18 |
| **1-5 years** | 134 | 1.08 | 55 | 1.22 | 39 | 1.93 | 40 | 0.68 |
| **5 years+** | 38 | 0.31 | 12 | 0.27 | 18 | 0.89 | 8 | 0.14 |
| **missing** | 117 |  | 14 |  | 39 |  | 64 |  |
| **family history of breast cancer** |  |  |  |  |  |  |  |  |
| **no** | 12289 | 98.31 | 4446 | 98.23 | 2009 | 97.67 | 5834 | 98.60 |
| **yes** | 211 | 1.69 | 80 | 1.77 | 48 | 2.33 | 83 | 1.40 |
| **missing** | 18 |  | 3 |  | 7 |  | 8 |  |
| **family history of cervical cancer** |  |  |  |  |  |  |  |  |
| **no** | 12296 | 98.34 | 4393 | 97.06 | 2040 | 99.08 | 5863 | 99.07 |
| **yes** | 207 | 1.66 | 133 | 2.94 | 19 | 0.92 | 55 | 0.93 |
| **missing** | 15 |  | 3 |  | 5 |  | 7 |  |
| **smoking** |  |  |  |  |  |  |  |  |
| **never** | 12452 | 99.50 | 4518 | 99.76 | 2012 | 97.67 | 5922 | 99.95 |
| **yes** | 62 | 0.50 | 11 | 0.24 | 48 | 2.33 | 3 | 0.05 |
| **missing** | 4 |  | 0 |  | 4 |  | 0 |  |
| **alcohol drinking** |  |  |  |  |  |  |  |  |
| **never** | 12167 | 97.23 | 4440 | 98.03 | 1836 | 89.13 | 5891 | 99.43 |
| **yes** | 347 | 2.77 | 89 | 1.97 | 224 | 10.87 | 34 | 0.57 |
| **missing** | 4 |  | 0 |  | 4 |  | 0 |  |
| **^a^ parous women only** | | | | | | | | |
| **^b^ postmenopausal women only** | | | | | | | | |

| **Table S2. Associations between selected characteristics and mammographic density** | | | |
| --- | --- | --- | --- |
|  | **BI-RADS c-d vs. a-b** |  | ***P*** |
|  | OR (95%CI) **^a^** |  |  |
| **age** |  |  |  |
| **per 2 years** | 0.81 (0.79-0.83) |  | <0.01 |
| **education** |  |  |  |
| **None-elementary** | 1 |  |  |
| **Middle school** | 0.90 (0.80-1.02) |  | 0.11 |
| **High school** | 0.95 (0.83-1.10) |  | 0.51 |
| **College** | 1.16 (0.98-1.37) |  | 0.08 |
| **BMI** |  |  |  |
| **per 2 kg/m^2^** | 0.75 (0.72-0.77) |  | <0.01 |
| **age at menarche** |  |  |  |
| **≤13** | 1 |  |  |
| **14-15** | 1.01 (0.91-1.13) |  | 0.83 |
| **16+** | 0.96 (0.86-1.07) |  | 0.44 |
| **parity** |  |  |  |
| **≤1** | 1 |  |  |
| **2** | 0.64 (0.57-0.71) |  | <0.01 |
| **3+** | 0.45 (0.38-0.53) |  | <0.01 |
| **age at first full term birth ^c^** |  |  |  |
| **≤20** | 1 |  |  |
| **21-24** | 0.95 (0.77-1.18) |  | 0.66 |
| **25-29** | 0.99 (0.79-1.24) |  | 0.94 |
| **30+** | 1.00 (0.72-1.39) |  | 1 |
| **Breastfeeding ^c^** |  |  |  |
| **No breastfeeding** | 1 |  |  |
| **1-6 months** | 0.80 (0.59-1.08) |  | 0.14 |
| **7-12 months** | 0.84 (0.66-1.07) |  | 0.16 |
| **13-18 months** | 0.76 (0.59-0.99) |  | <0.05 |
| **19-24 months** | 0.77 (0.60-1.00) |  | 0.05 |
| **25 months+** | 0.87 (0.67-1.13) |  | 0.28 |
| **menopausal status** |  |  |  |
| **premenopausal** | 1 |  |  |
| **postmenopausal** | 0.41 (0.36-0.45) |  | <0.01 |
| **age at menopause^d^** |  |  |  |
| **≤45** | 1 |  |  |
| **46-50** | 1.23 (1.06-1.44) |  | <0.01 |
| **51+** | 1.70 (1.43-2.02) |  | <0.01 |
| **family history of breast cancer** |  |  |  |
| **no** | 1 |  |  |
| **yes** | 1.05 (0.74-1.49) |  | 0.79 |
| ^a^ Binary logistic regression was used to estimate the ORs and 95%CIs comparing the dense (BI-RADS a-b) to non-dense breast group (BI-RADS c-d). adjusted factors: study site, age, education, BMI, age at menarche, parity, menopause status, family history of breast cancer | | | |
| ^c^ Parous women only. Model was additionally adjusted for age at first full term birth and breastfeeding | | | |
| ^d^ postmenopausal women only | | | |
